# Spatial Analysis of Childhood Cancer: A Case/Control Study

**DOI:** 10.1371/journal.pone.0127273

**Published:** 2015-05-20

**Authors:** Rebeca Ramis, Diana Gómez-Barroso, Ibon Tamayo, Javier García-Pérez, Antonio Morales, Elena Pardo Romaguera, Gonzalo López-Abente

**Affiliations:** 1 Environmental Epidemiology and Cancer Unit, National Centre for Epidemiology, Instituto de Salud Carlos III - ISCIII, Madrid, Spain; 2 Consortium for Biomedical Research in Epidemiology & Public Health (*CIBER en Epidemiología y Salud Pública*-*CIBERESP*), Madrid, Spain; 3 Public Health Division of Gipuzkoa, BIODonostia Research Institute, Department of Health of the regional Government of the Basque Country, Donostia, Spain; 4 Rare Disease Research Institute (Instituto de Investigación de Enfermedades Raras-IIER), Instituto de Salud Carlos III - ISCIII, Madrid, Spain; 5 Consortium for Biomedical Research in Rare Diseases (Centro de Investigación Biomédica en Red de Enfermedades Raras-CIBERER), Madrid, Spain; 6 Registro Español de Tumores Infantiles (RETI-SEHOP), Universidad de Valencia, Valencia, Spain; Institute for Health & the Environment, UNITED STATES

## Abstract

**Background:**

Childhood cancer was the leading cause of death among children aged 1-14 years for 2012 in Spain. Leukemia has the highest incidence, followed by tumors of the central nervous system (CNS) and lymphomas (Hodgkin lymphoma, HL, and Non-Hodgkin’s lymphoma, NHL). Spatial distribution of childhood cancer cases has been under concern with the aim of identifying potential risk factors.

**Objective:**

The two objectives are to study overall spatial clustering and cluster detection of cases of the three main childhood cancer causes, looking to increase etiological knowledge.

**Methods:**

We ran a case-control study. The cases were children aged 0 to 14 diagnosed with leukemia, lymphomas (HL and NHL) or CNS neoplasm in five Spanish regions for the period 1996-2011. As a control group, we used a sample from the Birth Registry matching every case by year of birth, autonomous region of residence and sex with six controls. We geocoded and validated the address of the cases and controls. For our two objectives we used two different methodologies. For the first, for overall spatial clustering detection, we used the differences of K functions from the spatial point patterns perspective proposed by Diggle and Chetwynd and the second, for cluster detection, we used the spatial scan statistic proposed by Kulldorff with a level for statistical significance of 0.05.

**Results:**

We had 1062 cases of leukemia, 714 cases of CNS, 92 of HL and 246 of NHL. Accordingly we had 6 times the number of controls, 6372 controls for leukemia, 4284 controls for CNS, 552 controls for HL and 1476 controls for NHL. We found variations in the estimated empirical *D(s)* for the different regions and cancers, including some overall spatial clustering for specific regions and distances. We did not find statistically significant clusters.

**Conclusions:**

The variations in the estimated empirical *D(s)* for the different regions and cancers could be partially explained by the differences in the spatial distribution of the population; however, according to the literature, we cannot discard environmental hazards or infections agents in the etiology of these cancers.

## Introduction

Childhood cancer was the leading cause of death among children aged 1–14 years for 2012 in Spain [1]. Among the 12 major groups of childhood cancer in the International Classification of Childhood Cancer third edition (ICCC-3) [2], leukemia has the highest incidence (aged adjusted rates per million children 0–14 years of age): Europe 44.0, Spain 47.0; followed by tumors of the central nervous system (CNS): Europe 29.9, Spain 33.2; and lymphomas: Europe 15.2, Spain 19.4 [3,4]. Causes for childhood cancer are mainly unknown with the exception of a small percentage of cases attributable to hereditary cancer syndromes (familiar retinoblastoma) or genetic syndromes and to exposure to ionizing radiation [5,6]. Early life exposure to environmental contaminants is suspected to be responsible for initial anomalies occurring in utero and leading to cancer [7]. Regarding leukemia many studies have addressed the hypothesis of infectious agents, but the association it is still not clear [8,9].

Spatial distribution of childhood cancer cases has been under concern in the last few decades [10–14]. In the nineties the EUROCLUS project for childhood leukemia analyzed the spatial distribution of 13351 cases diagnosed between 1980 and 1989 in 17 countries with the idea that the study of cluster and clustering could help to identify etiological factors. Their results indicated statistically significant evidence of clustering, but the magnitude was small [10,11,15]. A number of more recent studies have been conducted with this idea. A case-control study in California area showed no evidence of a non-random spatial pattern of childhood leukemia cases, although they have only 112 cases [14], two French studies with cases from the French National Register did not find statistically significant evidence of global heterogeneity of acute leukemia at small area level [12,16]; however, one study from the UK with data from the National Centre of Cancer Tumours found spatial clustering of leukemia in children aged 0–14 [17] and a second study found evidence of overall space-time clustering of childhood central nervous system tumors [13].

The study of the spatial distribution of cases can have two different purposes: one is overall spatial clustering analysis, which examines if the cases are closer to each other than the reference population; and the second purpose is cluster detection, the detection of a number of cases greater than expected in a specific geographical area. The objectives of this paper match these two purposes mentioned above. We studied overall spatial clustering and clusters of cases of the three main childhood cancer causes, looking to increase etiological knowledge.

## Materials and Methods

### Cases

**Fig 1:**
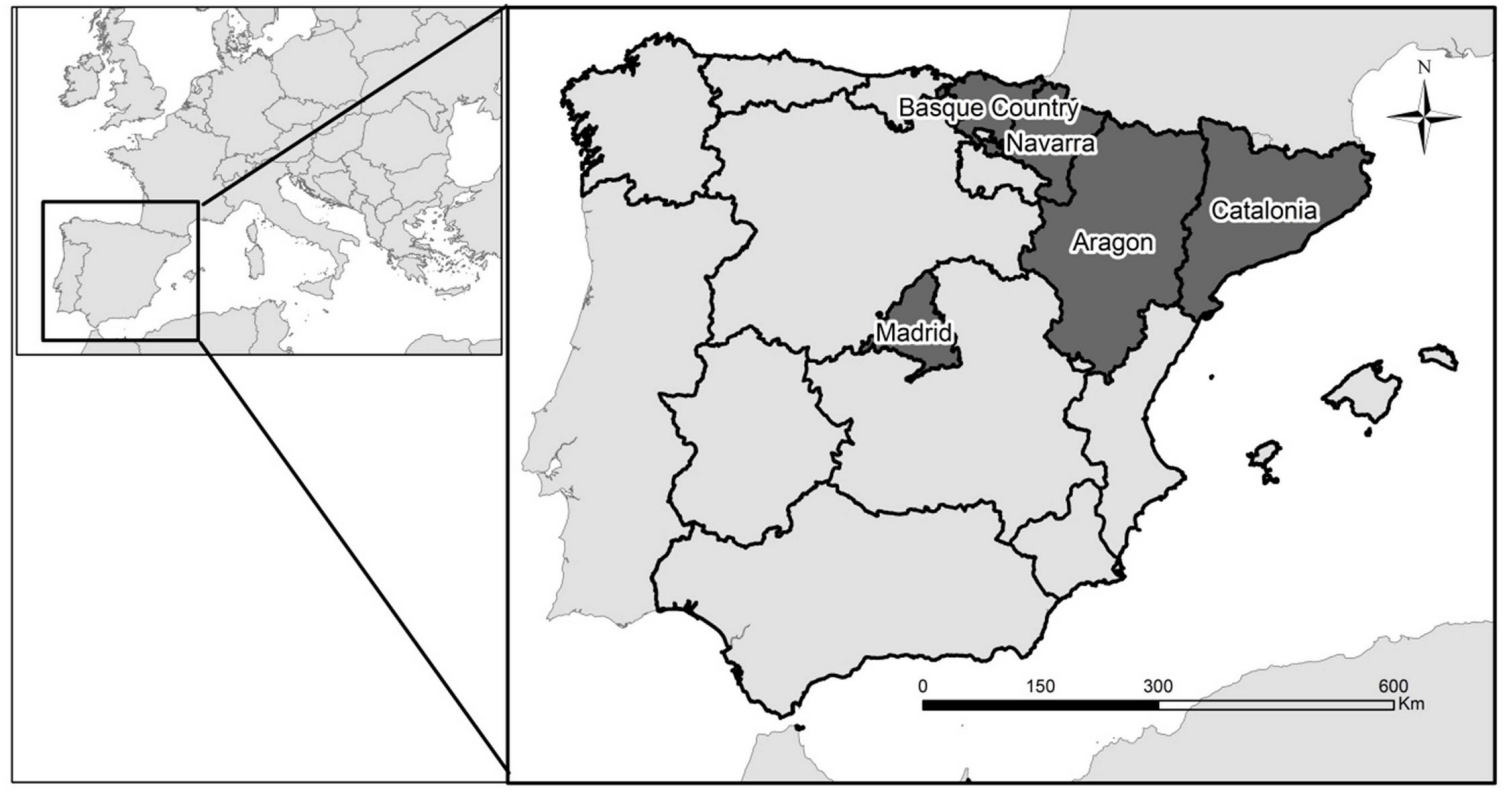
Map of Spain by regions. The regions included in the study are highlighted.

The Spanish Childhood Cancer Registry (RETI-SEHOP) collects information from all the pediatric oncology units in Spain and has the collaboration of the regional cancer registries. The completeness of the national coverage of childhood cancer by this registry is estimated at over 90% and 100% for the following five regions: Catalonia, Aragon, Navarre, the Basque Country and Region of Madrid [4]. The data used for the present study were children aged 0 to 14 diagnosed with a leukemia, lymphomas or CNS neoplasm, diagnostic groups I, II and III defined according to the 12 main diagnostic groups of the ICCC-3 [2]. For our analysis we separated the lymphomas into two groups, Hodgkin’s lymphomas (HL) and non-Hodgkin’s lymphomas (NHL). We included incidence cases from the five mentioned regions, four of them spatially contiguous located in the north-east part of Spain (North-East regions: Catalonia, Aragon, Navarra and the Basque Country) and an isolated one located in the center of Spain (Madrid). The studied period was 1996 to 2011 for all regions but Madrid, where the studied period was 2000 to 2011. (Fig 1 shows a map with the location of the regions).

Standard variables for each case included basic demographic data, such as date of birth, sex, province of residence and address at diagnosis. Also information on the diagnosis such as date, basis of diagnosis and morphology were included. We geocoded and validated the addresses of the cases by a geocoding strategy that is described later. We successfully validated 87% of the addresses. The remaining 13% of cases were fairly uniformly distributed along the different regions and therefore we did not think the data were biased in this sense.

### Controls

As a control group we used a sample from the population at risk extracted from the Birth Registry of the National Statistics Institute (Instituto Nacional de Estadística, INE). To select the controls we used a sampling strategy matching every case (with validated coordinates) by year of birth, region of residence and sex with six controls. Then we geocoded the addresses of the controls and we validated the coordinates. Only 2% of the controls did not have valid coordinates. Having had a small number of failures we decided to select more controls to replace this 2%, and we geocoded and validated this last group to end up with 6 controls with valid coordinates for every case.

### Geocoding strategy

For the purposes of this study, we conducted a retrospective geocoding (association of geographic coordinates from an input addresses) using the Google Map Javascript API v3. The obtained latitude and longitude data were projected into the ETRS89/UTM zone 30N (EPSG:25830) using QGIS software [18]. We then validated the coordinates and we kept those where the address and the coordinates matched. For the validation we performed the inverse process, we got the addresses of the obtained coordinates and we compared these new addresses to the original addresses. We compared town or city name, street name and street number.

### Methods

For our two objectives, overall spatial clustering analysis and cluster detection, we used two different methodologies. The first, for overall spatial clustering detection, was the differences of K functions from the spatial point patterns perspective proposed by Diggle and Chetwynd [19]. The second, for cluster detection, was the spatial scan statistic proposed by Kulldorff [20].

#### Overall spatial clustering analysis

Point patterns theory studies the spatial distribution of events occurring in a study region. The intensity of the phenomenon is the average density of points and it measures the ‘abundance’ or ‘frequency’ of the events recorded by the points. The intensity may be constant (‘uniform’ or ‘homogeneous’) or may vary from location to location (‘non-uniform’ or ‘inhomogeneous’). There are several methods to measure the intensity, one of them the K function proposed by Ripley [21]. The K function measures the aggregation of events at distance s and it is defined as:

$$K\left(s\right)\;=\;\frac{mean\;of\;events\;at\;distance\;\leq s\;from\;other\;event}{\;\lambda }$$

where λ is the density for the whole region.

Generally, the distribution of the population in space is inhomogeneous, especially the distribution of the population at risk when we study health events. To assess if the events (cases) are somehow spatially aggregated we need to compare their spatial distribution with the spatial distribution of the population at risk (controls). One way to do this is to compare the intensity of the cases and the controls by the comparison of their K functions. The method was proposed by Rowlingson and Diggle [22] and it is implemented in the Splancs library of R (21). They defined *D(s)* as the difference between *K(s)* for the cases and *K(s)* for the controls.

$$D\left(s\right)\;=\;K_{cases}\left(s\right)-K_{controls}\left(s\right)$$

The null hypothesis is *D(s) = 0*, not differences between the distributions. The distribution of *D(s)* under the null hypothesis is computed by a Monte Carlo simulation using random labeling. An envelope with the limits of the *D(s)* under the null hypothesis is also computed at the same time. While the empirical *D(s)* is between the limits of the envelope there is no evidence against the null hypothesis, only when *D(s*) is outside the envelope we can say than the spatial distribution of the cases is different to the spatial distribution of the controls. If the empirical *D(s)* is above the upper limit, we can say the cases are more aggregated than the controls and if the empirical *D(s)* is under the lower limit, controls are more aggregated than cases. We define as a maximum distance s equal to 8 km and we used the R software for the analysis [23].

#### Cluster detection

The spatial scan statistic is a test for spatial randomness based on likelihoods [20]. A cylindrical window that continuously changed its center and radius scanned the studied region seeking potential clusters. More precisely, the window moved from the address of a case to the address of another case. For each location the radius varied continuously from zero to a maximum distance (for our specific study we set a maximum of 5 km). Therefore, for each case the circular windows included different sets of neighboring cases and controls. For each location and size of scanning window the null hypothesis was that the risk was constant in space, the risk inside the window was the same that the risk outside. The alternative hypothesis was that the risk was higher inside than outside the window. During the process many different circular windows sides were evaluated in order to find the most likely cluster. Likelihood functions were calculated and maximized. The most likely cluster was the one with the maximum likelihood corresponding to a given location and specific radius. Its P value was obtained through Monte Carlo hypothesis testing (9999 replications), with a 95% confidence interval. Under the binomial assumption, the likelihood function for a specific window is proportional to:

$${{LR}_{i}\;=\;\left(\frac{n_{i}}{N_{i}}\right)}^{n_{i}}{\left(\frac{m_{i}}{M_{i}}\right)}^{m_{i}}I\left(\frac{n_{i}}{N_{i}}>\frac{m_{i}}{M_{i}}\right)$$

For each potential cluster *i*, *n*_*i*_ is the number of cases inside the potential cluster and *m*_*i*_ the number of cases outside, and *N*_*i*_ and *M*_*i*_ are the numbers at risk (cases and controls) inside and outside, respectively. *I()* is an indicator function that equals 1 when the risk inside the window is greater than the risk outside and 0 otherwise. We defined the level used for statistical significance as 0.05. Statistical analysis was performed with SaTScan 9.0.1 developed by Kulldorff [24]

#### Ethical considerations

Data used in this study are under protection by the Spanish law LOPD 15/1999 [25]. Privacy, confidentiality and rights of the cases and controls were ensured by changing the last digits of every coordinate (X and Y) by a random number.

## Results

After the geocoding and validation we had 1062 cases of leukemia, 714 cases of CNS, 92 of HL and 246 of NHL. Accordingly we had 6 times the number of controls, 6372 controls for leukemia, 4284 controls for CNS, 552 controls for HL and 1476 controls for NHL. For the analysis we separated the 4 Regions and Madrid, as we can see in Table 1 which shows the number of cases and control by cause and region. Table 2 shows a disaggregation of cases by cause, administrative region and sex.

**Table 1:** Number of cases and controls by cause and region.

Cancer site	Cases (Controls)North-East regions	Cases (Controls) Madrid
Leukemia	638 (3828)	424 (2544)
CNS	513 (3078)	201 (1206)
HL	53 (318)	39 (234)
NHL	144 (864)	102 (612)

**Table 2:** Number of cases by cause, region and sex.

Region	Sex	Leukemia	CNS	HL	NHL
North-East regions	M	369	273	38	106
	F	269	240	15	38
*Basque Country*	*M*	*76*	*42*	*6*	*11*
	*F*	*43*	*41*	*3*	*6*
*Navarra*	*M*	*23*	*23*	*1*	*7*
	*F*	*18*	*12*	*2*	*1*
*Aragon*	*M*	*34*	*30*	*4*	*13*
	*F*	*26*	*22*	*2*	*6*
*Catalonia*	*M*	*236*	*178*	*27*	*75*
	*F*	*182*	*165*	*8*	*25*
Madrid	M	247	111	30	71
	F	177	90	9	31

### Overall spatial clustering

**Fig 2:**
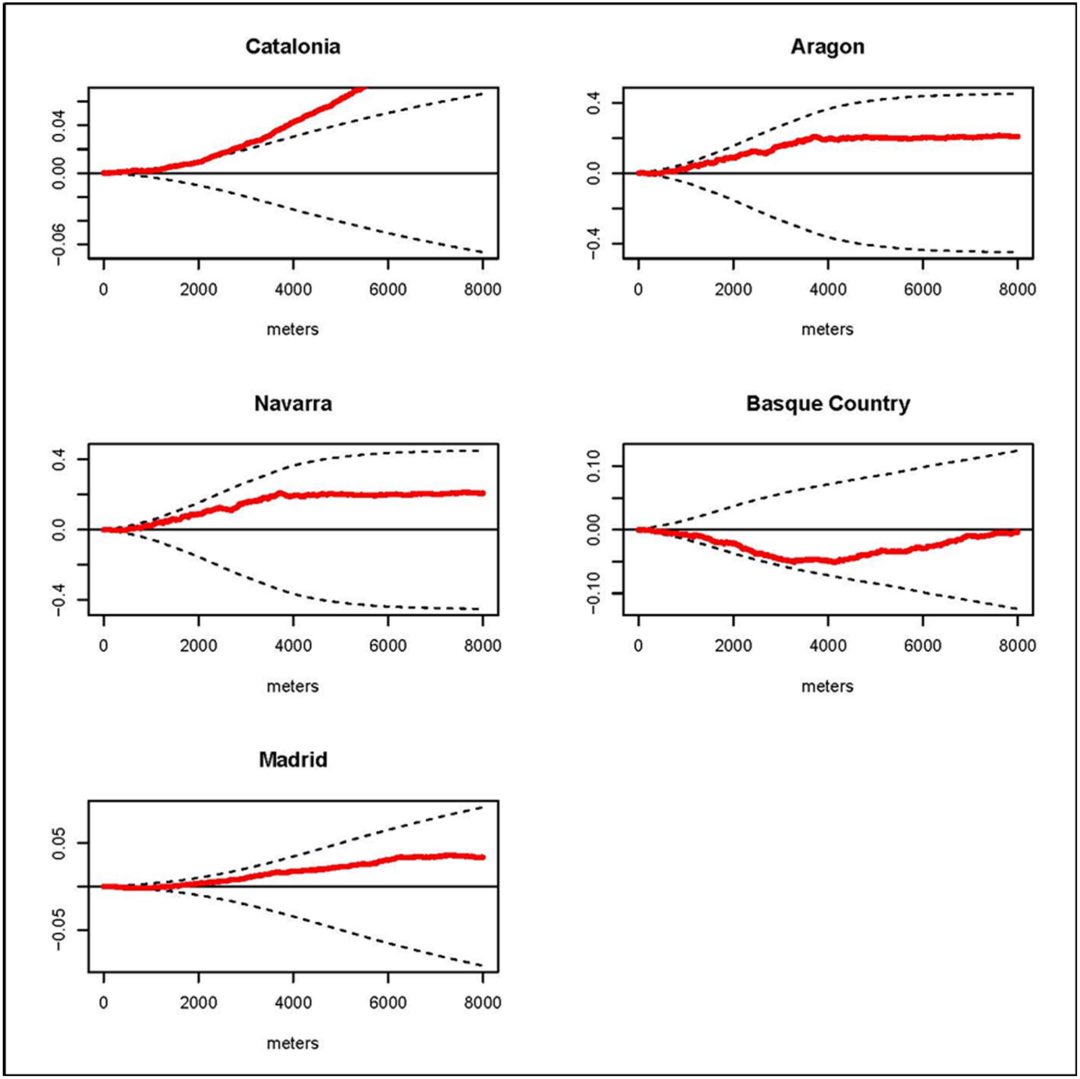
Leukemia. Graphs of the *D(s)* statistic function (red line) and the envelopes (dotted lines) from distance 0 to 8000 meters by region.

**Fig 3:**
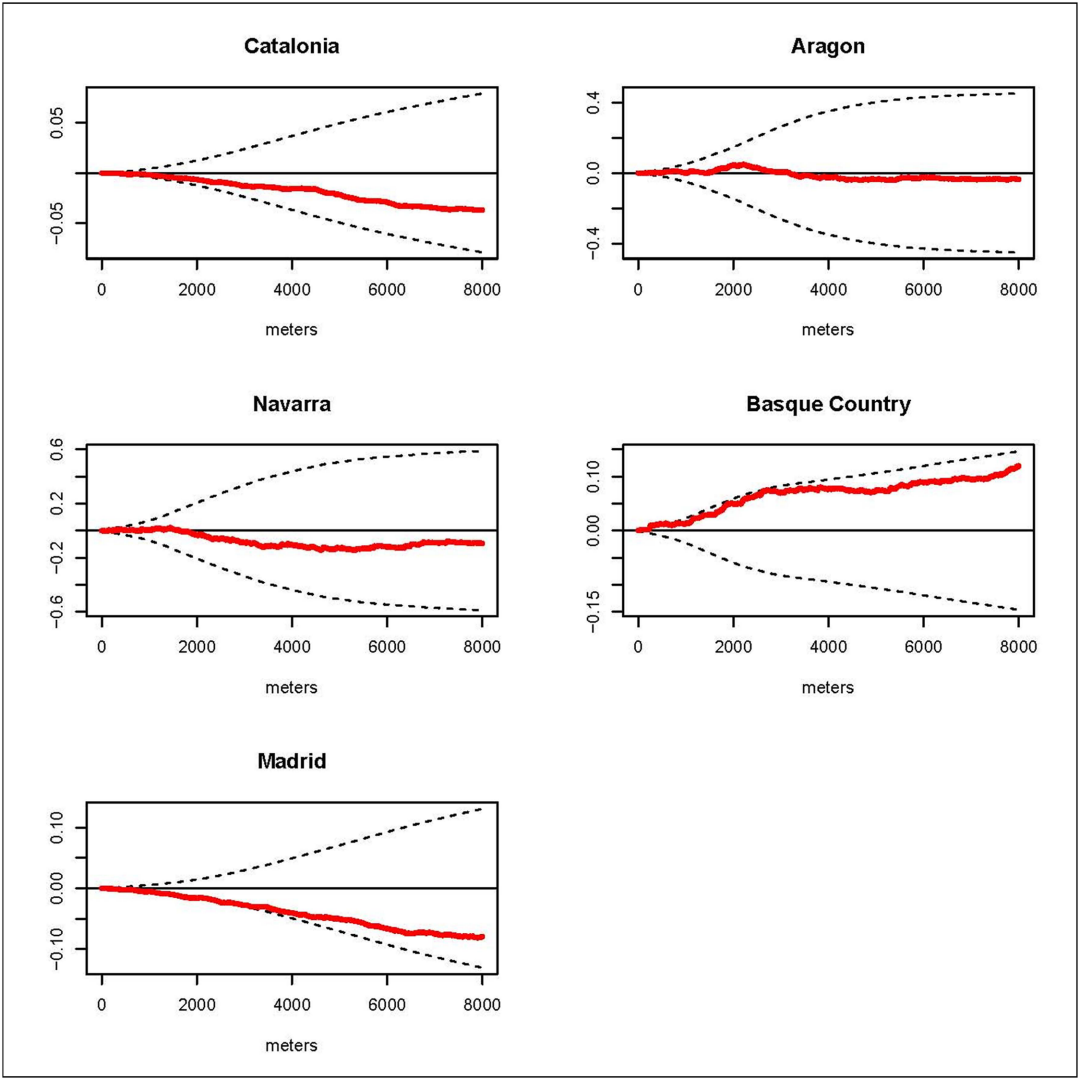
CNS. Graphs of the *D(s)* statistic function (red line) and the envelopes (dotted lines) from distance 0 to 8000 meters by region.

**Fig 4:**
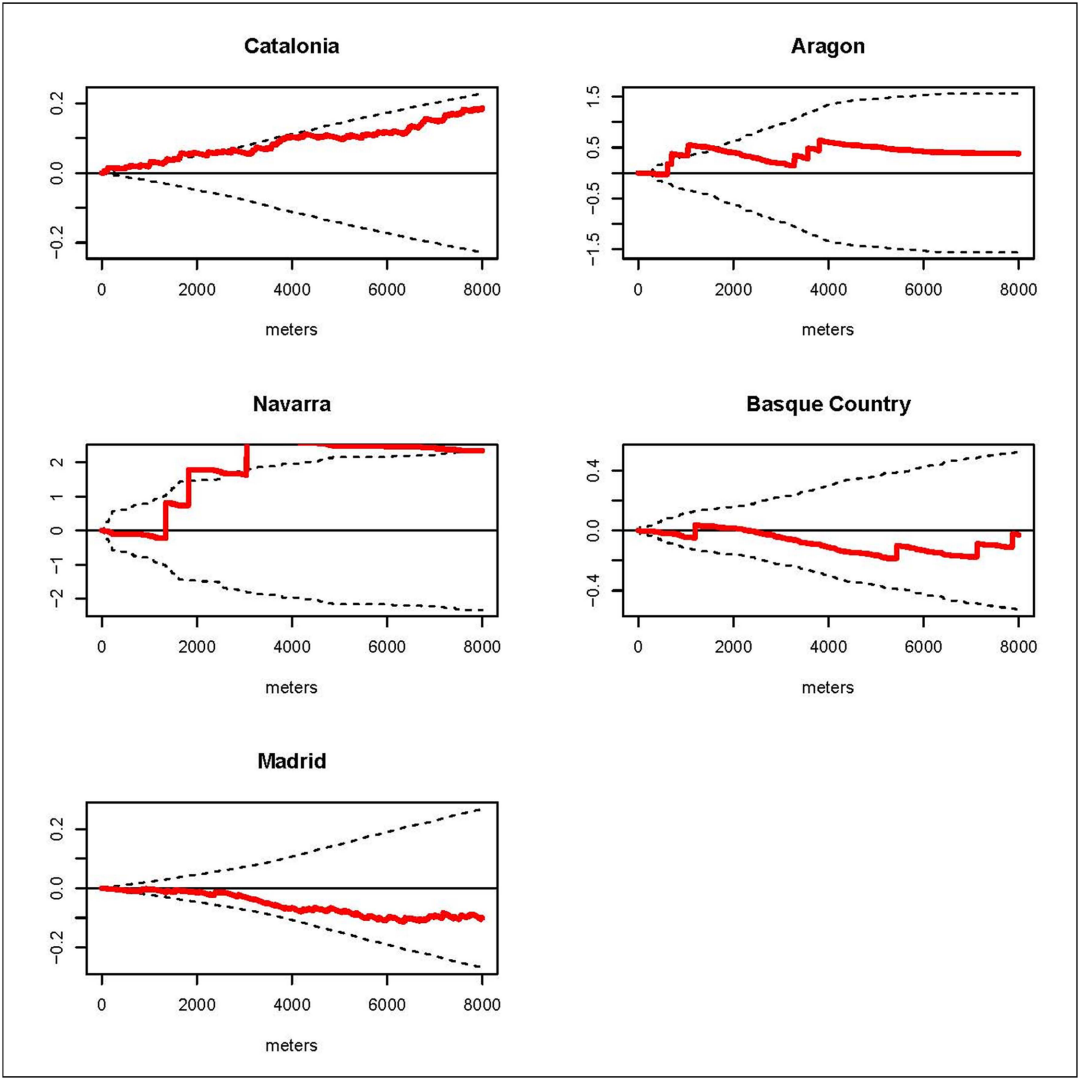
HL. Graphs of the *D(s)* statistic function (red line) and the envelopes (dotted lines) from distance 0 to 8000 meters by region.

**Fig 5:**
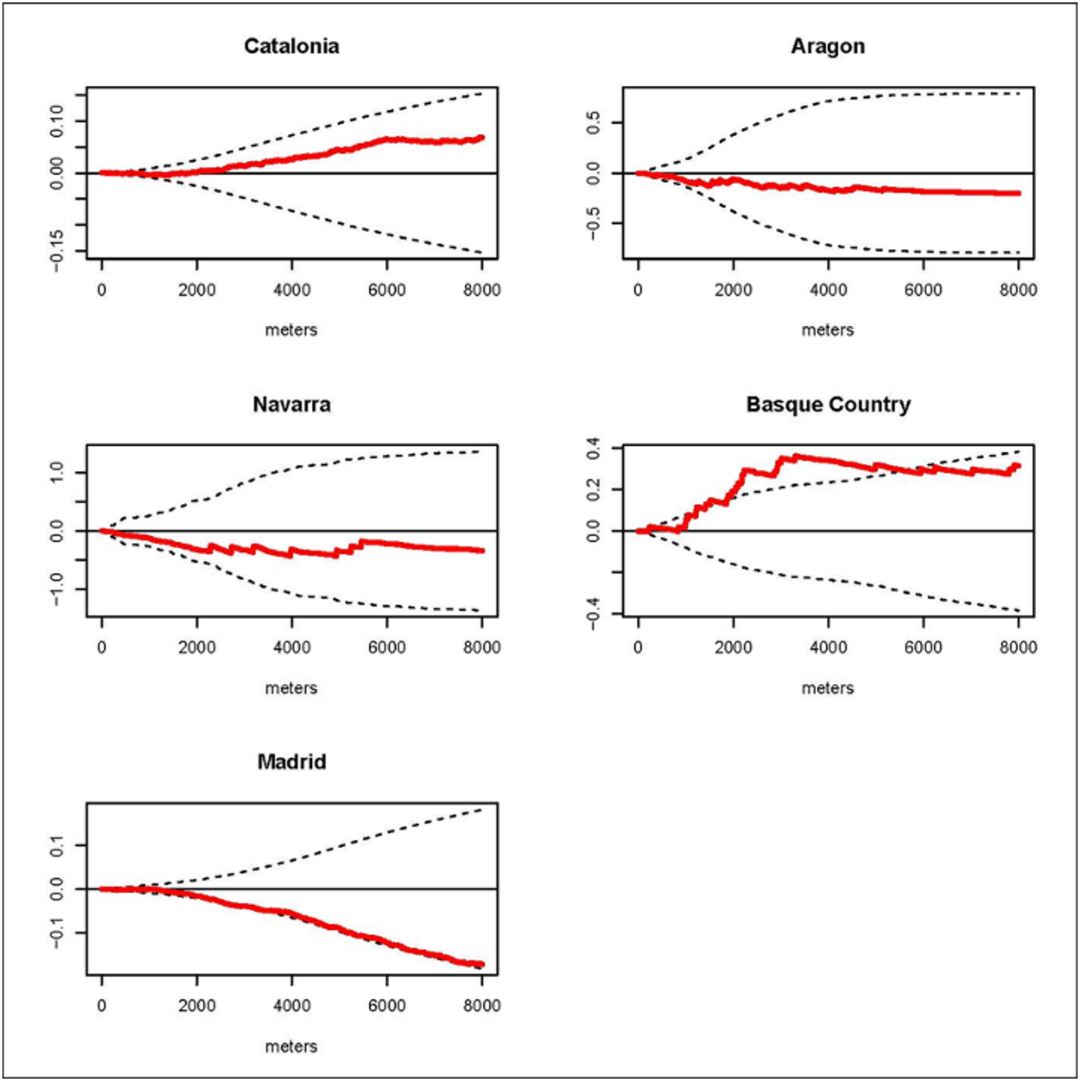
NHL. Graphs of the *D(s)* statistic function (red line) and the envelopes (dotted lines) from distance 0 to 8000 meters by region.

We estimated the *D(s)* statistic by region. The results for the K function by regions are shown in the graphs of the *D(s)* statistic included in Figs 2, 3, 4 and 5. These graphs show the evolution of the *D(s)* statistic versus the distance from 0 to 8000 meters. Generally, the empirical *D(s)* statistics are inside the envelopes for most of the causes, regions and distances *s*. However, for leukemia the empirical *D(s)* shows fairly regional variation and even for Catalonia it exceeds the upper limit from distance 3km and for the Basque Country the empirical *D(s)* statistic is closer to the lower limit. For CNS in the Basque Country the empirical *D(s)* slightly exceeds the upper limit for distances smaller than 1 km, and in Madrid it is very close to the lower limit. For HL the empirical *D(s)* shows obvious variations between the different regions: exceeding the upper limit from distance 0 to distance 2 km in Catalonia, from distance 1km to 2km Aragon, and for distances from 3km in Navarra. And for NHL the empirical *D(s)* exceeds the upper limit from distance 2 km to distance 6 km for the Basque Country, and in Madrid it is very close to the lower limit.

### Cluster

**Fig 6:**
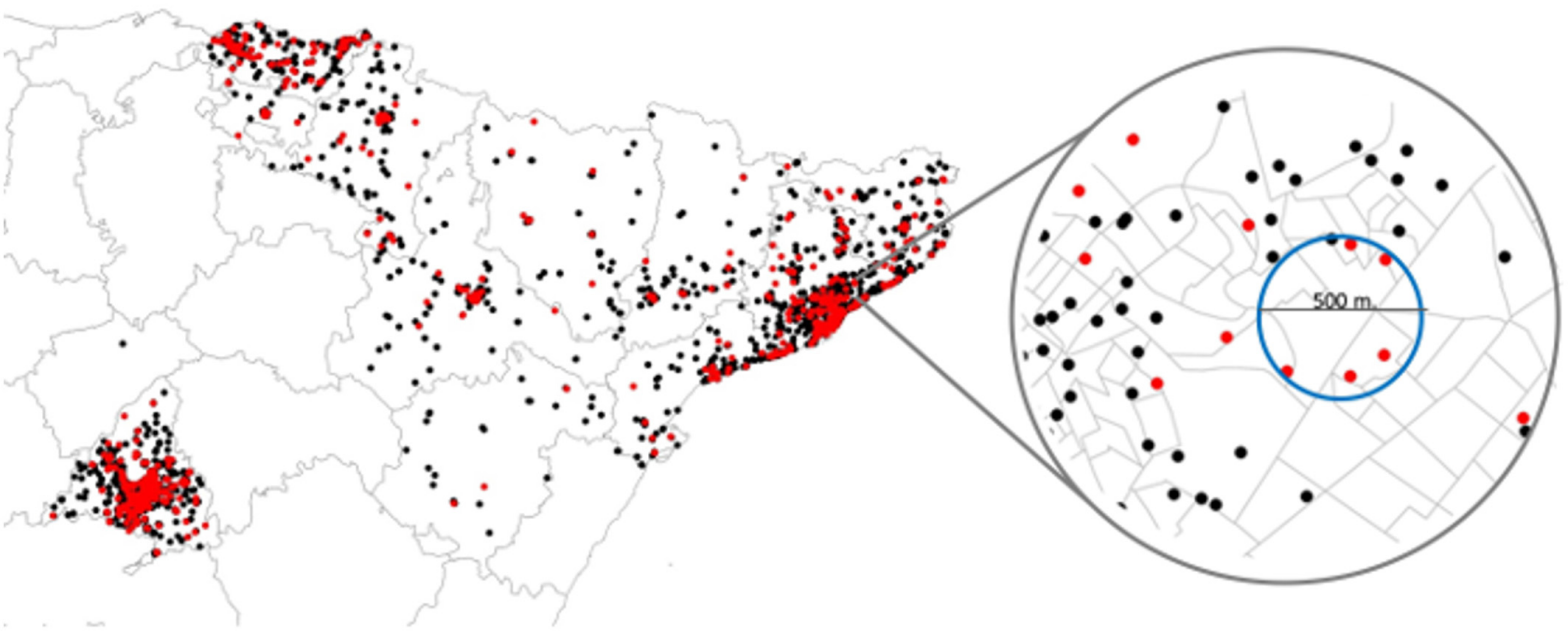
Map of leukemia cases and controls. Cases in red dots and controls in black dots. The right hand side shows a zoom to the primary cluster suggested by the scan statistic.

**Fig 7:**
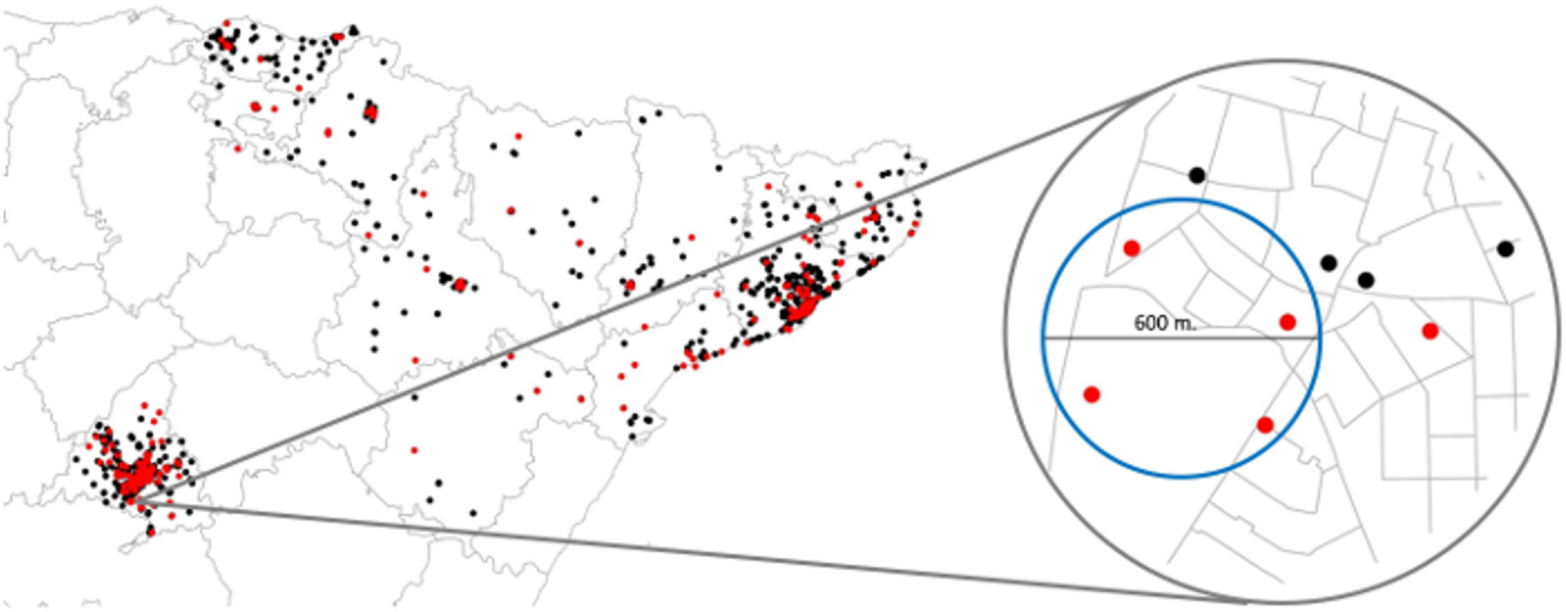
Map of NHL cases and controls. Cases in red dots and controls in black dots. The right hand side shows a zoom to the primary cluster suggested by the scan statistic.

The results from the spatial scan statistics are in table 3. We present the details for the primary cluster of every cause and region. There was no a statistically significant cluster detected. The lowest p-value (0.063) was for an aggregation of four cases of NHL in Madrid, and the second lowest (0.074) was for an aggregation of 5 cases of leukemia in Barcelona. Figs 6 and 7 show maps with the cluster of leukemia in Barcelona and the cluster of NHL in Madrid.

**Table 3:** SatScan results. Aggregation of cases with the lowest p-value by cause and region.

Cause	Region	Population	Cases	Expected cases	P-value	Radius (meters)	Location (Province)
Leukemia	NE regions	5	5	0.7	0.074	460	Badalona (Barcelona)
	Madrid	8	6	1.14	0.63	666	Colmenar Viejo (Madrid)
CNS	NE regions	20	10	2.86	0.92	886	Badalona (Barcelona)
	Madrid	9	6	1.29	0.54	1451	Madrid. Vallecas (Madrid)
HL	NE regions	3	3	0.43	0.122	124	Hospitalet de Llobregat (Barcelona)
	Madrid	2	2	0.29	0.54	573	Rivas Vaciamadrid (Madrid)
NHL	NE regions	3	3	0.43	0.48	619	Hospitalet de Llobregat (Barcelona)
	Madrid	4	4	0.57	0.063	643	Parla (Madrid)

## Discussion

This case-control study analyzes the incidence geographical patterns of the main childhood cancers, looking for overall spatial clustering and clusters of cases. For overall spatial clustering analysis generally there were no statistically significant differences in the spatial distribution of the cases and controls for leukemia, CNS tumors, HL and NHL in the studied regions. Nevertheless, we found clustering for leukemia cases in Catalonia at distances superior 3 km; for CNS in the Basque Country at distances smaller than 1 km; for HL in Catalonia up to distance 2 km, in Aragon from distance 1km to 2km and in Navarra for distances from 3km; and for NHL in the Basque Country from distance 2 km to distance 6 km. Generally there were variations in the estimated empirical *D(s)* for the different regions and cancers. Regarding cluster detection, we did not find statistically significant clusters. Nonetheless the results of the spatial scan statistic identified two aggregations of cases for leukemia and NHL with p-values close to 0.05. The locations of these detected potential clusters did not matchewith distances at which the empirical *D(s)* statistics were out of the envelopes.

Leukemias are the most frequent type of tumor in children and make up about the 30% of the cases [3,4]. According to Peris et al. the Spanish age standardized rate (ARS) (World standard population) for the period 1983–2002 was 45.93 cases per million [26]. The few well established risk factors are inherited cancer-predisposition and exposure to ionizing radiation but these factors account for only a few cases [27]. In our study leukemia is the tumor with more cases, 1062 in total. The results for the overall spatial clustering analysis did not show more aggregation in the cases than in the controls at any distance in any region but Catalonia for distances superior to 3 km. Nevertheless, the empirical *D(s)* showed fairly regional variation especially in the Basque Country and Catalonia in comparison to other 3 regions. The spatial scan statistics showed an aggregation of 5 cases with a p-value of 0.074 in Barcelona. The hypothesis of clustering in children with leukemia has been studied in the past. In the late nineties, the project EUROCLUS’s primary objective was to determine if the residence locations of cases at diagnosis showed a tendency towards spatial clustering; however, the results of the project could not confirm the hypothesis [11]. A case-control study in the San Francisco Bay area of the USA with 112 cases and 221 birth controls did not find evidence of non-random spatial patterns in the residences of the cases [14]. A French study of aggregated cases at area level (1916 areas for mainland France) did not find evidence of spatial heterogeneity either [12]. More studies have investigated links between exposure to environmental hazards and leukemia, a link that could show heterogeneity in the spatial pattern of the cases like those associated with exposure to pesticides [7]. Another etiological hypothesis that has been studied on many occasions is the exposure to infectious agents but its relation with leukemia is still unclear [8]. Our results did not show any spatial heterogeneity linked to environmental hazards, though this study was not designed with that aim in mind. Nonetheless, we cannot discard environmental hazards or infections agents in the etiology of leukemia in children based on our results, a spatio-temporal analysis could be more conclusive in this sense.

CNS tumors are the second most common cancer in children accounting for about 20% of the cases [3,4]. In Spain the estimated ASR for the period 1983–2002 was 32.83 cases per million [26]. For this study we had 714 cases of CNS tumors. Very little is known about the etiology of primary CNS and brain tumors. An estimated 5% of the cases may be explained by genetic predisposition, and the only established environmental risk factor is a high dose of ionizing radiation [28]. In our study CNS is the second most common tumor with 714 cases. For this cancer type neither of the two methods used suggested the presence of a pattern of overall spatial clustering or cluster, though the empirical *D(s)* for the Basque Country shows a tendency of clustering close to statistical significance. Results regarding the Basque Country and Navarra have special interest because these regions have showed higher incidence and mortality for CNS tumors at all ages [29]. On the other hand, several previous studies have found spatial patterns. A British study showed evidence of overall spatio-temporal clustering among cases of primitive neuroectodermal tumors [13]. Another British study found evidence of space-time clustering in cases of astrocytoma and ependymoma [30].

Lymphomas are the third most common cancer in children and make up around the 10–12% of the cases [3,4]. The estimated ASR for the period 1983–2002 for Spain was 18.48. For this study we separated the lymphomas into two groups: HL, with 92 cases, and NHL, with 247. HL in children is associated with the Epstein Barr virus [27]. For this lymphoma none of the two methods used suggested the presence of a pattern of spatial clustering or clusters, though the empirical *D(s)* is different for each of the 5 regions this could be due to the small number of cases. Little is known about the etiology of the NHL, but the associated genetic factors include congenital immunodeficiency syndromes [27]. Our results suggest no overall spatial clustering but the empirical *D(s)* is different for each of the 5 regions, and, again, this could be due to the small number of cases. There was a cluster of four cases of NHL in Madrid (p-value = 0.063).

One of the main strengths of our study is the large control group. Most studies of this type have one or two controls per case [14,31,32] in our study we have 6 controls per case and that gives a much more realistic image of the spatial distribution of the population at risk. Initially, we selected 6 controls as it is recommended by Rothman [33], since the georeference process was good and we got almost all the controls’ addresses’ coordinates, we decided to keep all of them and to replace the few that were missing with new controls.

The controls were randomly selected from birth certificates. This implies the possibility of having cases included in the control group, as excluding the cases as controls could bias the results [34]. The control group should give a clear view of the spatial distribution of the population at risk and should have the same risk of exposure as the cases. We matched the controls by sex, year of birth and region of residence to account for the temporal and regional variation in the child population. For the temporal trend there has been a moderate variation in the birth rate during studied period, starting with a birth rate of 1.15 for the year 1996 and reaching a maximum of 1.46 for year 2008 [1]. Regarding the regional variation, there are big differences between the regions; this study includes regions such as Madrid with a total population close to 6.500.000 inhabitants and Navarra with a total population of around 650.000 inhabitants for year 2012 [1]. This regional variation could be causing, to some extent, the variations observed in empirical *D(s)* for the different regions and causes.

It should be noted that we have the home address of the cases at the moment of diagnosis and the home address of the mother at birth for the controls. This difference could introduce bias in the analysis but according to official data, in Spain, only around 1% of the child population change their residence to a different province [1]. Therefore we considered that the home address at diagnosis is the same as the home address at birth for most of the cases.

We have used well-established methods for this analysis. For the overall spatial clustering analysis we used the method proposed by Diggle and Chetwynd for case-control studies. This method is based on the comparison of the second-order property of the observed point processes as a function of distance, a comparison made by the difference between the k functions [35]. The method has been used in different spatial analyses of epidemiological data [36,37] and it has an advantage above other methods in that the results show the specific distance at which the clustering occurs. The second method, Spatial scan statistics is a very popular method for cluster detection that has been used in many studies, primarily due to its availability in the free software package SatScan [24,35,38], its main flaw comes from the use of a regularly shaped window for the buffer [39].

A limitation for the study is that we performed the analysis across two disconnected areas, on one hand the north-east regions and on the other hand the Madrid region. However, this fact does not affect the results and conclusions. For overall spatial clustering we estimated the empirical *D(s)* for every region and cancer cause separately and then for cluster detection we defined 8 km as a maximum window. Consequently, we considered that the disconnection of the areas was not an issue. Another limitation is the lack of more information about potential exposure to risks factors for the children and their parents. The inclusion of these data in the analysis could provide more conclusive results.

## Conclusion

This study analyses the geographical patterns, overall spatial clustering and clusters, of individual incidence cases of childhood leukemia, CNS tumors, HL and NHL in Spain. We found spatial variation in the incidence of the main childhood cancers between the different regions and cancers, variation that could be partially explained by the differences in the spatial distribution of the population. Still according to the literature, we cannot discard the participation of environmental hazards or infectious agents in the etiology of these cancers.
